# How cognitive enhancement can change our duties

**DOI:** 10.3389/fnsys.2014.00131

**Published:** 2014-07-17

**Authors:** Filippo Santoni de Sio, Nadira Faulmüller, Nicole A Vincent

**Affiliations:** ^1^Department Values, Technology and Innovation, Faculty Technology, Policy and Management, Delft University of TechnologyDelft, Netherlands; ^2^Department of Experimental Psychology, University of OxfordOxford, UK; ^3^Department of Philosophy, Georgia State UniversityAtlanta, GA, USA

**Keywords:** cognitive enhancement, neuroenhancement, modafinil, methylphenidate, mental capacity, ethics, law, professional duties

## Abstract

This theoretical paper draws the scientific community’s attention to how pharmacological cognitive enhancement may impact on society and law. Namely, if safe, reliable, and effective techniques to enhance mental performance are eventually developed, then this may under some circumstances impose *new* *duties* onto people in high-responsibility professions—e.g., surgeons or pilots—to use such substances to minimize risks of adverse outcomes or to increase the likelihood of good outcomes. By discussing this topic, we also hope to encourage scientists to bring their expertise to bear on this current public debate.

## Introduction

Whereas techniques for the augmentation of brain function are usually seen as beneficial when used as a form of medical treatment, both amongst the general public (Schelle et al., 2014) and in academia (e.g., Sandel, 2009) some concerns have been raised about the possible negative moral and social impacts of the use of these techniques in healthy people. For example, concerns about such so called “cognitive enhancement” include seeing it as a threat to the fairness and meaningfulness of competitive activities, or as a potential threat to a meaningful human life. Even though “cognitive enhancement” may refer to different brain intervention techniques like genetic modification, pharmacological substances, Transcranial Magnetic Stimulations (TMS), or Transcranial Direct Current Stimulation (tDCS; Bostrom and Sandberg, 2009), in this paper we focus on *pharmacological* cognitive enhancers such as methylphenidate and modafinil. These substances have been reported to modestly improve wakefulness, attention, concentration, learning and retention of memory, not only when taken by people diagnosed with mental deficits or disorders, but also when taken by healthy individuals (Repantis et al., 2010; Husain and Mehta, 2011; Coffman et al., 2014; Gilleena et al., 2014; Meinzer et al., 2014).

In what follows, we discuss a seldom-recognized but crucial way in which pharmacological cognitive enhancement may impact on our society’s moral and legal norms: the availability of such enhancers might evoke *new* *duties* for certain people. In particular, it may impact on the *professional duties* of people engaged in jobs where the lives of other people are directly at risk (e.g., surgeons and pilots)—i.e., it may impact on what we can (legally) *demand* these professionals to do. By exploring this issue, we want to offer some insights into a particular way in which scientific work on brain function augmentation may impact on society: by enhancing our cognitive capacities, neuroscientific progress may change our duties to one another (for in-depth discussion see Vincent, 2011, 2013; Enck, 2014; Goold and Maslen, 2014; Santoni de Sio et al., 2014).

## How pharmacological cognitive enhancement can create new duties

Our main question may be framed in the following way: assuming that a certain kind of pharmacological enhancement proves to be relatively safe and effective at reducing risks of negative outcomes, may some people, in virtue of what is at stake in the performance of their professional roles (e.g., surgeons or pilots), be sometimes *legitimately expected* to cognitively enhance themselves even if they would rather not do so? Even though this question may sound counterintuitive at present, we think there are good reasons to assume that such an expectation might be a realistic scenario in the future. In particular, we think that the professional duty to use pharmacological enhancers may sooner or later be raised in tort cases at least under restricted circumstances, for instance in emergencies, when less invasive and at least as effective alternatives are not available. If this happens, judges will take a decision mainly through analogical reasoning. We thus think it is important to anticipate how such reasoning is likely to run. By having a clearer picture of such a possible future scenario, the scientific community and the public more generally will be able to think ahead about whether any pre-emptive steps need to be taken to forestall the development of foreseeable undesirable social, political, and legal consequences involved in such a scenario.

In the following we present three main points in support of our claim that a duty to enhance, as circumscribed above, may arise in the future.

Firstly, scientific and technological progress has already affected professional duties in the past. Surgeons, for instance, are nowadays expected to deploy many measures that enhance their performance and/or reduce the risks of fatal outcomes. Historically, this professional duty to take measures has gradually emerged over time, and progress in scientific and technological knowledge has been one decisive element in the creation of the duty. When, for instance, basic antiseptic procedures which are common today—e.g., cleansing hands with carbolic acid—were originally developed, their efficacy was not yet established, their risks for the user were unknown, they were available only in select research laboratories and medical practitioners were not expected to deploy them. But today, now that the clinical value of these techniques is widely recognized, and they are relatively inexpensive, largely free of risk, and ubiquitously available (Gawande, 2012), medical practitioners cannot legitimately reject the request to employ these techniques. The discovery of the antiseptic efficacy of carbolic acid, as it were, brought with it the creation of a duty to use it.

Of course, the analogy between cognitive enhancement medications and carbolic acid is far from perfect—while the former is highly invasive to an important domain, namely brain functioning, the latter is not even skin-deep. Therefore, one should not expect this analogy to be sufficient to make a case for the professional duty to use pharmacological enhancers. However, this analogy is arguably sufficient to make a more general point: when it comes to professions with a high societal value like those aimed at healing people or warranting their safety, it may be legitimate for society to demand professionals to not follow their individual preferences but rather the rules of good practice that are proven to lead to optimal results, including those requiring them to undertake particular treatments of their body. Even in the most democratic society, the value of individual freedom of choice of professionals is not protected unconditionally. For instance, at present we already expect medical or legal professionals to engage in continuing education programs. Often this is quite an invasion on people’s lives since they must set aside time from an often already busy schedule to attend classes after hours, often losing sleep, and certainly losing personal time. But yet we do not think that this imposition on their freedom is an unreasonable one. We think that this is a sacrifice that we are entitled to expect professionals to make for the benefit of their patients and clients. The underlying thinking is that what’s gained in terms of outcomes is presumed to be more important than the sacrifices that others have to make to secure those outcomes.

Secondly, it may certainly be insisted that things are different with pharmacological enhancers, and that no matter what happens with the compulsory use of non-invasive technologies or with compulsory non-pharmacological enhancement programs, the use of medical substances that directly affect the brain can never be imposed on professionals against their will. However, even as things currently stand we already sometimes expect some people to use medical substances that directly affect their brain for the benefit of others—namely, when we expect people who wish to operate motor vehicles but who are diagnosed with conditions like epilepsy and diabetes to take medical substances to prevent the negative effects of these conditions from adversely affecting others (*Knoxville Optical Supply, Inc. v Thomas*, 1993 WL 574 (TennCtApp Jan 04, 1993)). Naturally, a good reason for them to take these medications is simply the benefits to their own health. But it has to be noted that the argument which justifies legal coercion to use those substances in the case of the epileptic and diabetic motor vehicle drivers is not that the medical substances will benefit *them—*in a liberal democracy paternalism is rarely accepted as a valid justification for infringements on freedom—but rather that their not taking those medications would impose an unacceptable risk to *others* (if they should take to the roads un-medicated).

We offer the above example in support of two points. Firstly, there is already an existing and accepted practice of expecting one group of people to take brain-invasive medications for the benefit of other groups of people. Secondly, it makes little difference that these examples involve the use of medications to *treat* rather than to *enhance*, because the persons concerned are expected to take the medications not for their own benefit, but for the benefit of others. In fact, Queensland Health, the medical regulatory body of the North-East Australian state, has recently followed a similar reasoning pattern in relation to fatigue management. In their Queensland Health (2009), it is suggested that in order to cope with fatigue-related risks, surgeons could take up to “400 mg of caffeine [which is the] equivalent to about 5–6 cups of coffee” (78) because “[c]ompared with other psychoactive drugs (e.g., modafinil), caffeine is… more readily available and less expensive” (79). Given that the report explicitly cites modafinil, and that it only cites availability and cost as considerations that favor the use of caffeine over modafinil, we think it is perfectly conceivable that a future report may recommend such drugs to be used (cf. Maslen et al., in press). To be sure, one may still insist that this is not a desirable scenario, and that the protection of minds from external interference should be recognized as a human right. However, as a matter of fact, the right to “cognitive liberty” (Bublitz, 2013) is not (yet) protected by international human rights in the same way in which bodily integrity is, so that the scenario that we propose remains realistic.

Finally, one may wonder whether concerns about safety will in the end prevent the imposition of a duty to enhance in every situation imaginable. Admittedly, the long-terms possible negative effects of the use of cognitive enhancers are not sufficiently known (Madras et al., 2006; Volkow et al., 2009), and a greater scientific understanding of these substances is needed before normative conclusions can be confidently drawn (Maslen et al., 2014). However, it is also a fact that methylphenidate has been prescribed to children to treat the symptoms of attention deficit and hyperactivity disorder for well over two decades. We cite this example only to illustrate that there is at present already consensus about the relative safety of these drugs to prescribe them to the most vulnerable part of the population—namely, to children. This point is salient because if, as a society, we deem the costs or risks of particular cognitive enhancement technologies to be sufficiently low, then this may lead us, together with the other considerations mentioned above, to impose a duty to use these medications onto some people at least in some emergency situations, namely when other more common forms of intervention like napping or being replaced by another worker who is not fatigued are not available.

## A glance into the future

Admittedly, we are not (yet) in the scenario that we just described. The efficacy of pharmacological cognitive enhancement techniques in reducing the rate of fatal mistakes and thus enhancing the quality of performance of professionals like surgeons and airline pilots has not been established yet (Förstl, 2009; Repantis et al., 2010), and they are not even easily accessible. It is therefore not surprising that the law has not yet demanded any professionals to enhance themselves nor have lay people advanced such a demand.

As for the law, Goold and Maslen (2014) have offered a detailed legal analysis on the issue of whether surgeons who are at risk of making fatigue-related errors during patient care might be considered legally obliged to pharmacologically enhance themselves, i.e., if, at least under certain circumstances, there can be a *legal duty to enhance* for surgeons. Their conclusion is that, at the moment, such a legal duty *cannot* be imposed (at least in England and Wales). However, once one considers the reasons behind their conclusion, it becomes clear that Goold and Maslen’s statement about the current legal situation is not necessarily incompatible with our claim about the future. Their case against the imposition of a legal duty to enhance on surgeons, in fact, critically depends on their reasonable doubts about the efficacy of current enhancers, and the possible negative side-effects of these substances. Moreover, this conclusion does not affect the validity of our theoretical point. In fact, as Goold and Maslen themselves explicitly state, in a *hypothetical* scenario in which efficacious and relatively safe cognitive enhancers were available, surgeons *might* be burdened with a duty to take pharmacological cognitive enhancers to reduce the risks of fatal fatigue-related error, at least under some emergency circumstances, namely when other, less invasive, options like napping or being replaced by another surgeons are not available. And it is *this* kind of hypothetical scenario that our reasoning has taken into account, and that future judges may have to decide upon.

As for lay people, they also seem to believe that no obligation to enhance should ever be imposed on professionals or other subjects (Maslen et al., in press). Many are indeed skeptical even about the moral *permissibility* of the use of pharmacological enhancers in any circumstance (Santoni de Sio et al., in press). Again, this is reasonable and understandable. Lay reasoning widely reflects the current state of affairs of scientific progress. From this perspective, lay reasoning is somehow similar to that of the above-mentioned legal scholars: because pharmacological cognitive enhancement is currently neither uncontroversially efficacious nor safe, people are legitimately wary and suspicious of it and reluctant to expect anyone to use it. However, lay reasoning may also reflect less rational and justified concerns. Medical enhancement substances are perceived negatively compared to other “natural” enhancers, and as a consequence the use of pharmacological cognitive enhancement might be stigmatized also in an irrational way (Faulmüller et al., 2013).

## Conclusion

Reflections on the social impact of scientific and technological progress can come in different forms. On the one hand, we may reflect on how *current* technologies and techniques are already impacting on society. However, we may also wish to reflect on how the social, political, legal, and moral landscape may change due to pressure from reasonably expected *future* advances in science and technology, and think ahead about whether any pre-emptive steps need to be taken to forestall the development of foreseeable undesirable social, political, legal, and moral consequences. Adopting this latter “socially responsible innovation” approach (Moor, 2008; van den Hoven, 2013) which has recently been embraced also by the European Commission (2011), our paper has discussed the possible impact on society of future advances in pharmacological cognitive enhancement. We have argued that the availability of techniques that can enhance performance may in the future impose *new* *duties* on certain people under certain circumstances.

## Conflict of interest statement

The authors declare that the research was conducted in the absence of any commercial or financial relationships that could be construed as a potential conflict of interest.
